# Dichotomy effects of Akt signaling in breast cancer

**DOI:** 10.1186/1476-4598-11-61

**Published:** 2012-08-24

**Authors:** Zhengang Peng, Jennifer Chao Weber, Zhaosheng Han, Rulong Shen, Wenchao Zhou, James R Scott, Michael WY Chan, Huey-Jen L Lin

**Affiliations:** 1 Division of Medical Technology, School of Allied Medical Professions, The Ohio State University, Columbus, OH, 43210, USA; 2 Molecular Biology and Cancer Genetics Program, Comprehensive Cancer Center, The Ohio State University, Columbus, OH, USA; 3 Department of Pathology, The Ohio State University, Columbus, OH, USA; 4 College of Medicine, University of Arizona, Phoenix, AZ, USA; 5 Department of Life Science and Human Epigenomics Center, National Chung Cheng University, Min-Hsiung, Chia-Yi, Taiwan, ROC; 6 Department of Medical Laboratory Sciences, University of Delaware, Room 305 Willard Hall Education Building, 16 West Main Street, Newark, Delaware, 19716, USA

**Keywords:** Activated Akt signaling, Breast epithelia, Epithelial-mesenchymal transition, Motility, Stem-progenitor cells

## Abstract

**Background:**

The oncogenic roles contributed by the Akt/PKB kinase family remain controversial and presumably depend on cell context, but are perceived to be modulated by an interplay and net balance between various isoforms. This study is intended to decipher whether distinct Akt kinase isoforms exert either redundant or unique functions in regulating neoplastic features of breast cancer cells, including epithelial-mesenchymal transition (EMT), cell motility, and stem/progenitor cell expansion.

**Results:**

We demonstrate that overactivation of Akt signaling in nonmalignant MCF10A cells and in primary cultures of normal human mammary epithelial tissue results in previously unreported inhibitory effects on EMT, cell motility and stem/progenitor cell expansion. Importantly, this effect is largely redundant and independent of Akt isoform types. However, using a series of isogenic cell lines derived from MCF-10A cells but exhibiting varying stages of progressive tumorigenesis, we observe that this inhibition of neoplastic behavior can be reversed in epithelial cells that have advanced to a highly malignant state. In contrast to the tumor suppressive properties of Akt, activated Akt signaling in MCF10A cells can rescue cell viability upon treatment with cytotoxic agents. This feature is regarded as tumor-promoting.

**Conclusion:**

We demonstrate that Akt signaling conveys novel dichotomy effects in which its oncogenic properties contributes mainly to sustaining cell viability, as opposed to the its tumor suppressing effects, which are mediated by repressing EMT, cell motility, and stem/progenitor cell expansion. While the former exerts a tumor-enhancing effect, the latter merely acts as a safeguard by restraining epithelial cells at the primary sites until metastatic spread can be moved forward, a process that is presumably dictated by the permissive tumor microenvironment or additional oncogenic insults.

## Background

The serine/threonine kinase Akt/PKB has emerged as one of the most pivotal protein kinase family that plays critical roles in regulating pleiotropic cellular and physiological processes [1]. In response to ligand stimulation from cytokines or from growth factors such as the insulin-like growth factor-I (IGF-I) and the epidermal growth factor (EGF) family, receptor tyrosine kinases are phosphorylated, an event which subsequently activates phosphatidylinositol 3-kinase (PI3K) signaling and stimulates the Akt axis as well as other downstream signaling pathways [1]. To commence this signaling cascade, the lipid second messenger phosphatidylinositol (3,4,5)-triphosphate (PIP3) is first synthesized from PIP2 by PI3K and then recruits both Akt and phophoinositide-dependent kinase 1 (PDK1) to the plasma membrane through the pleckstrin homology (PH) domain where the highly conserved Thr308 is phosphorylated by PDK1 [2,3]. Phosphorylation of this residue as well as Ser473, catalyzed by the mammalian target of rapamycin complex 2 (mTORC2), together confers full activation of Akt [4], thereby activating various downstream factors by phosphorylating arrays of targets [1]. Conversely, this activation cascade can be blocked by cellular inhibitors including the phosphatase and tensin homolog (*PTEN*) and *INPP4B* which directly antagonize PI3K function *via* dephosphorylating PIP3, thereby abrogating PIP3-mediated activation of downstream signaling events such as PDK1 and Akt [5,6]. As a result, target cells can be returned to a basal level in a standby mode. However, *in vitro* engineered Akt kinase can override this regulatory mechanism and maintain it in a “supercharged” stage. This can be done by insertion of myristoylated (Myr) tag at its N-terminus which results in anchoring Akt in plasma membrane anchorage as well as constitutive activating Akt independently of PI3K activity [7,8].

Three main Akt isoforms, Akt1/PKBα, Akt2/PKBβ, and Akt3/PKBγ, have been discovered in mammals and they share two highly conserved regions in the PH and kinase catalytic domain [1]. Studies employing gene ablation in laboratory animals have revealed non-redundant functions of Akt isoforms. *Akt1* null mice are small with significant growth defects [9,10], whereas mice depleted of *Akt2* develop insulin-resistant diabetes [11]. Conversely, *Akt3* ablation leads to reduced brain size in mice [12,13]. Notably, differences in physiology and microenvironment might exist between mice and humans. Clinical studies demonstrated that specific isoforms can be amplified in different types of human cancer, furthering a notion that Akt kinase dictates transformation phenotypes of various carcinomas in an isoform-specific manner, rather than in a redundant fashion [14]. Yet, how etiological cause(s) determine which of the 3 isoforms shall be activated and subsequently transmit unique downstream targets to exert distinct outcomes remains largely unknown.

Furthermore, components within the PI3K pathway are frequently dysregulated in human cancers [15]. For instance, activating mutations of *PIK3C* (the catalytic subunit of PI3K) often occur in prevalent carcinomas [16]. Moreover, *PTEN* has been proven to be one of the most commonly altered genes in human malignancies [17]. In contrast, gain-of-function Akt mutations are relatively uncommon [15,17] and most frequently occur at residue 17 (namely E17K) which resides in the PH domain and is thus unlikely to directly sustain kinase activation. Based on clinical studies, it is becoming doubtful that Akt activation *per se* is indeed important for driving various neoplastic phenotypes. In support of this notion, activated Akt signaling was previously shown to induce senescence as well as inhibit breast cancer cell motility and invasion [18-21].

Among its known neoplastic features, Akt kinase is involved in EMT, which is characterized by the loss of epithelial characteristics and the acquisition of a mesenchymal phenotype [22]. In carcinomas, EMT is associated with increased aggressiveness, tumor invasion, and metastatic potential, and endows mammary stem cell properties [23-25]. A recent study demonstrated that Akt activation *via* down-regulated PTEN can enrich normal as well as malignant human mammary stem/progenitor cells and these aberrations can be rescued by Akt inhibitors [26]. Nevertheless, a mounting body of evidence supports the idea that Akt signaling regulates cell migration and EMT *via* an isoform-specific and context-dependent manner [27-30]. It remains largely unclear whether Akt kinase would result in different outcomes, in respect to normal *versus* malignant breast epithelia. Moreover, it remains puzzling as to whether Akt activation augments a whole array of transformation phenotypes collectively leading to oncogenesis, or if it exerts paradoxical effects on both promoting and impeding neoplastic phenotypes.

To investigate these issues, we have expressed all three isoforms of constitutively active Myr-Akt kinase in human mammary epithelia ranging from nonmalignant primary epithelia, an immortalized cell line, and a series of cell lines exhibiting varying degrees of malignant behavior. This wide array of target cells has allowed us to reveal how Akt influences oncogenic phenotypic changes corresponding to the cell context in varying degrees of malignancy. We have discovered that Akt, in an isoform-independent fashion, has tumor suppressive properties since it can inhibit of EMT, decrease cell motility, and reduce the stem/progenitor cell population. These aberrations are rather prominent in non-malignant epithelia but diminish as cells progress to a more neoplastic state. However, even in non-malignant cells, Akt activation can have tumor-promoting properties since it can promote cell survival following exposure to chemotherapeutic agents. Taken together, this study denotes a novel paradigm that activated Akt signaling can have both tumor-suppressing and tumor-promoting properties.

## Results

### Activated Akt signaling impedes EMT and attenuates cell migration in non-malignant breast epithelia

Our preceding report revealed that, in non-malignant breast epithelial cell line such as MCF10A, Akt signaling can be activated by tumor microenvironmental stimuli provoked from an exposure to breast cancer-associated fibroblasts [31]. However, it remains rather controversial how Akt signaling affects breast oncogenesis since data generated from animal models is inconsistent with data from clinical studies [28,30,32], despite the fact that various isoforms may display distinct and opposing effects [27-30]. Herein, we assessed the effects of activated Akt signaling on neoplastic behavior in human breast epithelia.

**Figure 1:**
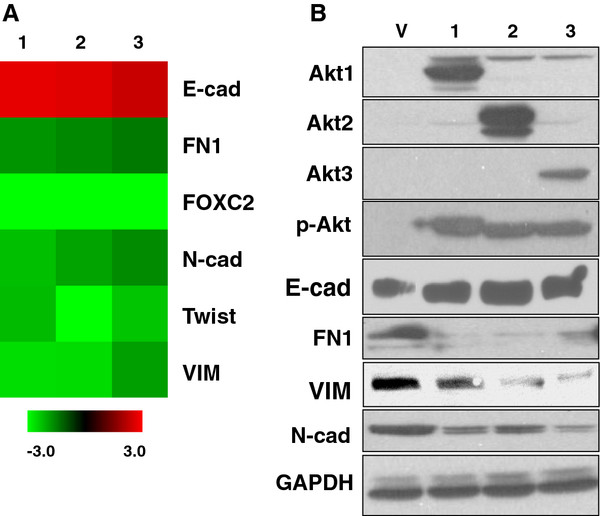
**Activated Akt signaling, regardless of isoform type, attenuates expression of EMT-associated transcripts and proteins in MCF10A cells. A.** Cells were infected with either control retrovirus Babe (V) or with one expressing Akt1 (1), Akt2 (2) or Akt3 (3), respectively. The total RNA was extracted from the respective cells and subjected to RT-qPCR to quantify the transcripts of interest. Heatmap represents relative mRNA levels of EMT-associated transcripts in Akt-infected cells compared to the ones in control counterpart. **B.** MCF10A cells were lyzed and the resultant mixture was subject to Western immunoblotting using indicated antibodies to assess the levels of proteins of interest. Similar level of GAPDH expression was an indicative of nearly equal amounts of proteins introduced in all samples.

**Figure 2:**
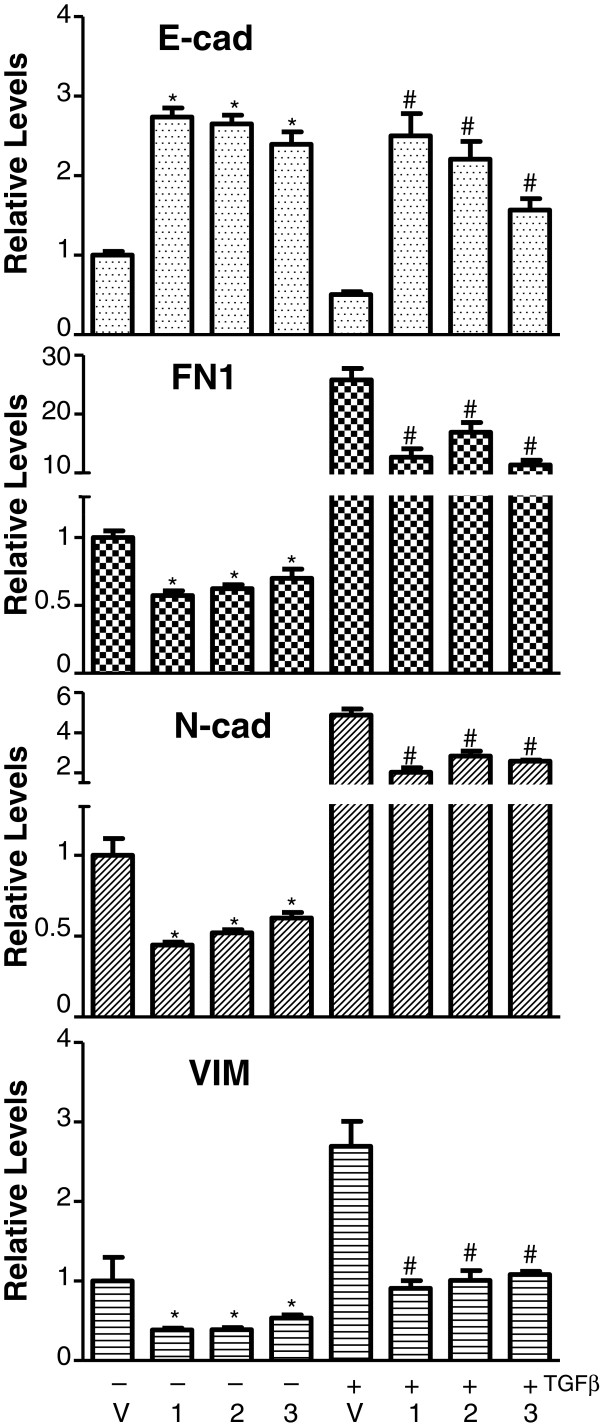
**Activated Akt inhibits TGFβ-induced EMT in an isoform-redundant manner.** MCF10A cells were infected with retroviruses to express empty vector (V), Akt1 (1), Akt2 (2) or Akt3 (3) and then cultured in the absence or presence of TGFβ (2 ηg/ml, R & D research) for 3 days followed by RT-qPCR to analyze EMT-associated transcripts. Significant changes in the experimental groups compared to vector control were marked with * or with # (respectively denoting without or with TGFβ treatment) if *p* < 0.05.

**Figure 3:**
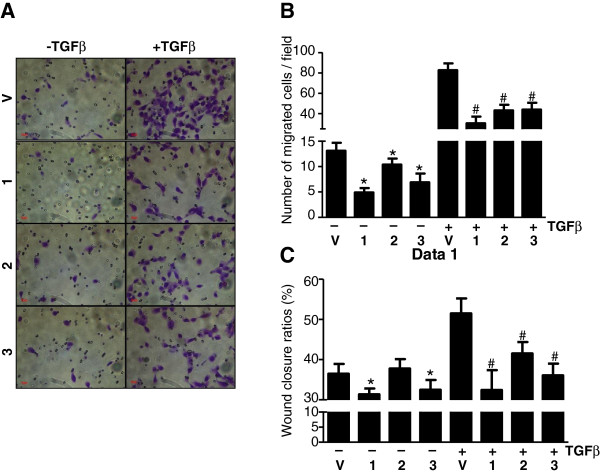
**Akt signaling represses basal motility as well as TGFβ-induced migration in an isoform-independent manner.** MCF10A cells expressing vehicle control (V), Akt1 (1), Akt2 (2) or Akt3 (3) were either untreated or stimulated with TGFβ for 3 days. The motility of the resultant cells was assessed either by migration assay at 12-hours (**A** and **B**) or wound healing assay 20-hours later (**C**).

As breast cancer mortality is largely ascribed to metastatic spread that is tightly related to EMT and cell motility, the influence of Akt activation on these aberrations is of great interest. Hence, constitutive expression of Akt was engineered by transducing Myr-Akt *via* retroviral-delivery system into MCF10A cells. Two weeks later when maximal expression and Akt kinase activity was reached (data not shown), total RNA was extracted from the resultant cells and subjected to RT-qPCR assays to quantify the expression levels of a panel of known EMT transcripts, including the epithelium-associated protein E-cadherin (E-cad) as well as the mesenchymal associated proteins fibronectin (FN1), FOXC2, N-cadherin (N-cad), Twist, and Vimentin (VIM) [22,33]. Interestingly enough, regardless of isoform types, activated Akt signaling consistently yielded a noticeable induction of E-cad along with an inhibition of various mesenchymal-associated transcripts (Figure 1A; and the left panel of Figure 2). Western blotting confirmed that the changes in mRNA levels are also seen at the protein level (Figure 1B). This observed suppression of EMT is mirrored by a moderate decrease in cell motility, as measured by using transwell migration and wound-healing scratch assays (left panels of Figure 3A, 3B; and 3C). In these experiments, activation of either Akt1 or Akt3 resulted in a greater than 2-fold inhibition of motility compared to vehicle controls, whereas activation of Akt2 resulted a less prominent effect. Nevertheless, our finding indicates that none of the AKT isofoms were able to promote mesenchymal properties nor enhance cell mobility in nonmalignant MCF10A cells, implicating a potential tumor-repressing rather than tumor–promoting role as indicated in previous reports [18-21].

Furthermore, to exclude the likelihood that our observation is due to the fact that MCF10A cells are immortalized, this discovery was further substantiated by using non-immortalized primary normal human mammary epithelial cells (HMEC) isolated from three different women. Similar to the results obtained using the MCF10A cells, activation of Akt inhibited the expression of mesenchymal-associated transcripts (Additional file 1: Figure S1A) and reduced cell motility (Additional file 1: Figure S1B) in HMECs from all three donors. These effects were not associated with any specific Akt isoform, with the exception that expression of E-cad was marginally repressed in HMEC-2 overexpressing Akt3 as well as in HMEC-3 expressing all 3 isoforms (Additional file 1: Figure S1A). Likewise, N-cad was largely inhibited in HMEC-1 and -2, but activated in HMEC-3. We view outliers as being probably ascribed to an induction of another unreported adhesion molecule(s) that can trigger an epithelial phenotype *in lieu* of E-cad activation or N-cad repression. The variability might be due to the fact that commercial primary breast epithelia are rather heterogeneous compared to MCF10A cells. Nevertheless, migration was prominently inhibited by activated Akt signaling (Additional file 1: Figure S1B). Taken together, for the first time by studying all three Akt isoforms, our data suggest that overly activated Akt signaling can result in a noticeable reduction of mesenchymal-associated transcripts as well as a decrease in cell motility and these are observed in non-malignant breast epithelial cells including not only immortalized MCF10A (Figures 1, 2 and 3) but also primary breast epithelial cultures HMEC (*n* = 3) (Additional file 1: Figure S1).

### Activated Akt signaling hinders IGF-I and TGFβ-induced EMT in an isoform-independent manner

The Akt pathway axis has been reported to be modulated by distinct isoforms [27,29]. Most functional studies of Akt isoforms have been performed *via* gene-specific xknockdown of specific Akt isoforms in genetically modified mice. However, the latter is limited by species conservation and potentially biased by the fact that tumor-microenvironment in the mouse may not always reflect the that in humans [34]. The discrepancy of data evolved from the two system might be ascribed to ectopic expression (current study) *versus* knocking down endogenous Akt [27,29]. However, knocking down specific Akt isoforms seems to be less relevant than overexpression strategies since human carcinomas frequently display aberrant activation and amplification rather than suppression of Akt signaling [17].

**Figure 4:**
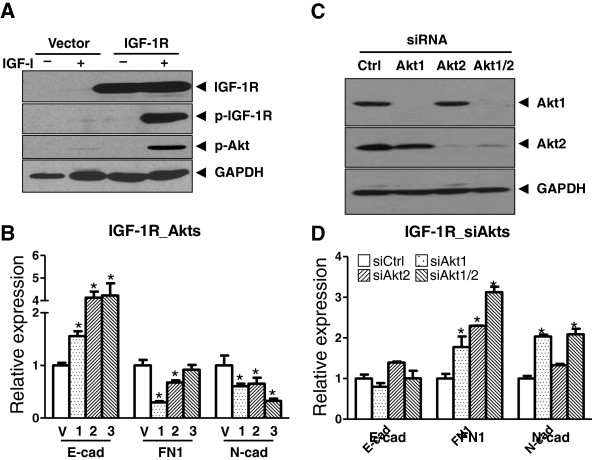
**Activated Akt inhibits IGF-1 induced EMT in an isoform-independent manner. A.** Vector control or IGF-1R-expressing MCF10A cells were either left untreated or treated with IGF-I (100 ng/mL) for 72 hours and then analyzed by Western immunoblot using indicated antibodies. Authentication of IGF-1R expression was evidenced by an elevated phosphorylation of IGF-1R after IGF-I treatment. **B.** IGF-1R-expressing MCF10A cells were infected with retrovirus expressing either vehicle control (V), or Akt1 (1), -2 (2), or -3 (3) and then subsequently treated with IGF-I for an additional 3 days, followed by RT-qPCR analysis for assessing EMT-associated transcripts. Statistical significance was denoted by * if the transcripts in Akt-expressing cells significantly (*p* < 0.05) differed from the ones in vector control cells (V). **C.** Knocking down endogenous Akt by transfecting IGF-1R cells with siRNAs targeting either Akt1 or Akt2 or in combination were evidenced by a prominent loss of Akt expression assessed by Western blots. **D.** In IGF-1R-exprssing MCF-10A cells pre-treated with IGF-1, knocking down Akt rescue altered EMT-associated transcripts influenced by IGF-1R signaling (as compared to panel B).

To decipher how different Akt isoforms influence IGF-I-mediated EMT, MCF10A cells were retrovirally-transduced to express IGF-IR that subsequently became phosphorylated and activated (Figure 4A), which in turn induces EMT [27] in response to ligand stimulation. However, we observed that ectopic expression of any isoform of constitutively activated Myr-Akt largely attenuated the EMT shift induced by IGF-IR stimulation since we detected an increase of E-cad transcripts and a reduction of FN1 and N-cad transcripts (Figure 4B). This observation was further supported by another experiment in which knockdown Akt1 or Akt2 alone or in combination by siRNA resulted in an opposite effect (E-cad, FN1, and N-cad; compare Figure 4D to 4B). Noticeably, siRNA knockdown of Akt exerted a less prominent effect on E-cad expression. We speculate this outcome might be due to compensatory effects provoked from aberrant pathways that are influenced by a loss of Akt signaling. It is known that expression of E-cad can be regulated by various signaling pathways including interleukin-4 [35], Interleukin-15 [36], miR-34a induced by hypoxia [37], ERK-MAPK pathway triggered by C-Met signaling [38], and Wnt-signaling cascade induced by leptin [39]. We hypothesize that knocking down Akt by siRNA unexpectedly results in perturbations in these pathways, and this subsequently restores E-cad expression that is otherwise suppressed (Figure 4D).

To confirm the suppressive effects of Akt signaling on EMT, we also examined the ability of Akt signaling to reverse TGFβ-induced EMT. Treatment of MCF-10A cells with 2 ηg/ml TGFβ induces EMT. However, overexpression of any Akt isoforms can decrease transcripts associated with EMT (Figure 2) and can decrease cell motility in transwell migration as well as wound-healing scratch assays (Figure 3). Taken together, our data suggest an unreported finding that, in an isoform-independent manner, overly activated Akt can result in an inhibitory effect on EMT induced by IGF-IR or by TGFβ in non-malignant breast epithelial cells.

The molecular mechanisms responsible for down-regulating TGFβ-induced EMT features by Akt were investigated further. We first examined epigenetic regulation mediated by regional occupancy of various core nucleosome proteins. Post-translational modifications, including methylation, acetylation, phosphorylation, or ubiquitination, occurring at various residues laying in the N-termini of histone proteins can result in either up- or down-regulation of target gene expression [40]. “Histone code” works by either changing the accessibility of chromatin or by recruiting and/or occluding non-histone effector proteins to regulate transcriptional activities [40]. By examining aberrant histone occupancy on a panel of promoters of loci associated with EMT, we discovered that occupancy of dimethylated lysine 4 on histone H3 (H3K4me2), indicative of a transcriptionally active chromatin, at the *VIM* promoter region was lower in Akt-overexpressing MCF10A than the one in vector control cells when the cells were treated with TGFβ (Additional file 1: Figure S2). Interestingly enough, this effect closely paralleled the finding generated from both transwell migration and wound-healing scratch assays (Figure 3), indicating that reduced occupancy of H3K4me2 at *VIM* promoter might be correlated with Akt-mediated inhibition of EMT and cell motility.

### Akt signaling reduces stem/progenitor subpopulations in normal breast epithelia

**Figure 5:**
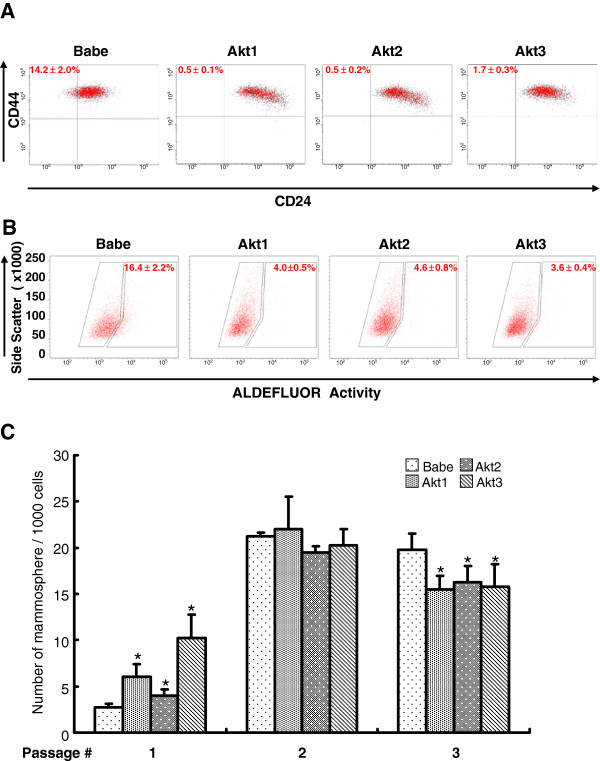
**Activated Akt signaling decreases breast stem/progenitor cell subpopulations.** MCF10A cells were infected with retroviruses to respectively express empty vector (Babe), Akt1, Akt2, or Akt3 and then subject to PE-CD24 and FITC-CD44 double-staining (**A**), ALDEFLUOR assay (**B**), or serially passaged mammosphere formation assay from which mammospheres were counted under a microscope by independent investigators (**C**). * Denotes that the number of mammospheres obtained from Akt-expressing cells differed significantly (*p* < 0.05) from the vector controls.

EMT, as well as overexpression of HER2, activation of PI3K, and loss of PTEN have all been associated with acquisition of stem/progenitor cell properties [23,24,26,41-43]. Since we demonstrated thatMyr-Akt was able to inhibit EMT (Figures 1, 2, 3, 4), we decided to investigate if Akt also inhibited the acquisition of the stem cell state. To do this, we manipulated Akt expression in MCF-10A cells and then measured the frequency of cells expressing a stem/progenitor cell phenotype (CD44^+^/CD24^-/low^, ALDH^+^) as well as having the ability to generate mammospheres in non-adherent cultures. As shown in Figure 5A, MCF10A cells expressing vehicle control (denoted as Babe) had approximately 14% of cells with a CD44^+^/CD24^-/low^ phenotype, which is consistent with previous reports [44,45]. However, activation of respective Akt1, Akt2 or Akt3 uniformly decreased the frequency of this subpopulation to 0.5 ~ 1.7% of total cells (Figure 5A). Likewise, this inhibitory effect mirrored the data generated by ALDEFLUOR assay where the proportion of ALDH^+^ cells decreased from ~16% (in Babe control cells) to 3.6 ~ 4.6% (in cells overexpressing activated Akt) regardless of Akt isoform type (Figure 5B). Our finding that activated Akt represses stem cell fractions is consistent with other reports demonstrating that constitutive active Akt depletes hematopoietic stem cell populations [46]. All proper negative controls for gating cells for the flow cytometric assays are described in Additional file 1: Figures S3 and S5.

To confirm that Akt overexpression decreases the stem/progenitor cell population using a functional assay, cells overexpressing Akt as well as control cells were seeded into the mammosphere assay and the frequency of cells that can generate spheres was determined. Results demonstrate that Myr-Akt expression was able to reduce the frequency of mammosphere-initiating cells in the parent populations compared to the vector controls (passage 3, Figure 5C). It is noteworthy to mention that this inhibitory effect was not noticeable until later passages. For example, activated Akt expression enhanced mammosphere formation during passage 1, whereas it exerted marginal changes during passage 2. We interpret this contradictory outcome as being due to Akt’s intrinsic property of facilitating anchorage-independent growth [8], an effect that might interfere with the current test analyzing mammosphere formation. However, this biasing effect gradually diminished over a couple of passages and became a less interfering factor during passage 3 (Figure 5C). Consequently, such biasing factor might have underscored the extent of inhibiting mammosphere formation that, otherwise, would have been comparable to the CD44^+^/CD24^-/low^ and ALDH^+^ phenotype data (Figure 5A and 5B). Taken together, the effect of inhibiting stem/progenitors closely concurs with our data describing the suppressive effects of Akt activation on EMT transcripts and cell motility (Figures 1, 2, 3, 4, 5).

### Malignant state of breast epithelia dictates the EMT inhibitory effects exerted from Akt signaling

**Figure 6:**
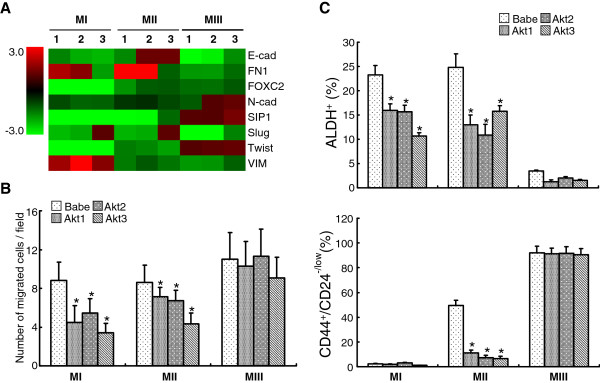
**The inhibitory effects of Akt signaling on EMT, cell motility and stem/progenitor cell subfraction can be attenuated in cells that become progressively malignant. A.** MI, MII and MIII cells were infected with retroviruses expressing vector control (Babe), Akt1 (1), Akt2 (2), or Akt3 (3), followed by RT-qPCR analysis for assessing expression levels of EMT-associated transcripts. Heatmap represents relative mRNA levels of various transcripts in Akt-expressing cells compared to the ones in control cells set as 1. **B.** Infected MI, MII, or MIII cells were tested for migration assay 12 hours after seeding on the top chamber of transwell plate and * denotes a significant change (*p* < 0.05) as compared to the control cells (Babe). **C.** Infected MI, MII or MIII cells were subjected to ALDEFLUOR staining (upper) or PE-CD24 plus FITC-CD44 double-staining (lower), followed by FACS analysis to quantify the frequency of ALDH^+^ and CD44^+/high^/CD24^-/low^ subpopulations. * denotes significant (p < 0.05) difference between the Akt-expressing cells and the counterpart control.

Discrepancies among published findings regarding the oncogenic roles Akt plays suggest a possibility that Akt signaling might exert differential effects that are related to the degree of malignancy of the cells and the cellular context [27-30]. To investigate this further, the current study was expanded to include a set of isogenic cell lines (MI, MII and MIII) that were derived from MCF10A cells, but later underwent a step-by-step oncogenic transformation so that a series of lines with progressively increased tumorigenicity was generated (from the lowest MI to the highest MIII) [47]. Similar to MCF10A and HMEC cells, pre-malignant MI and MII cells, but not MIII, displayed generally reduced expression of EMT-hallmarked transcripts (Figure 6A) along with a declined motility (Figure 6B) in response to activated Akt signaling. In general, aberrations were more prominent in MI than in MII, implying an inverse correlation between inhibitory effects and the malignant states of the cells. Along the same notion, the suppressive effect became diminished in the highly malignant cell line MIII (Figure 6A and 6B) and thereby indicating that Akt-mediated inhibitory effects are likely to be blocked when cells have undergone advanced transformation. It is possible that additional oncogenic pathways embedded in MIII might have cross-talked with and thus liberated the inhibitory effects provoked by Akt signaling.

The notion that advanced neoplastic features can combat the inhibitory effect exerted from Akt activation was substantiated by evaluating the influence of Akt on maintenance of stem/progenitor cell populations in the isogenic cell line system. Despite the fact that the intrinsic stem/progenitor subpopulations differ among MI, MII and MIII (Figure 6C, vector control, Babe), overexpression of any of the three Akt isoforms concordantly repressed the frequency of ALDH^+^ cells with mean inhibition rates being > 40% in MI and > 50% in MII (Figure 6C, upper panel), but rather negligible in MIII. As MIII harbors a nearly undetectable ALDH^+^ subpopulation (nearly 2%), the necessity of assessing the CD44^+^/CD24^-/low^ phenotype became apparent. Interestingly, we observed that the fraction of CD44^+^/CD24^-/low^ cells is proportional to the malignant state (around 2%, 50% and 90% in MI, MII and MIII cells, respectively) (Figure 6C, lower panel). Although the inhibitory effect of Akt on MI was undetectable due to its extremely low basal level, MII was influenced to a remarkable degree (from ≥ 50% in Babe control to ≤10% in Akt-expressing cells)(Figure 6C, lower panel). In sharp contrast, this inhibitory effect was completely blocked in MIII cells (Figure 6C, lower panel). Moreover, this rescuing effect is in close agreement with data generated from the transwell migration assay (Figure 6B). Together, they depict a novel paradigm that Akt-mediated inhibition of EMT transcripts, cell motility, and stem/progenitor cell expansion, is perhaps inversely associated with the malignant status of breast epithelia. Most importantly, this idea can be recapitulated in advanced breast cancer cell lines (BrCa-MZ-01 and SUM159) in which Myr-Akt expression rendered undetectable inhibitory effects on sustaining the ALDH^+^ subpopulation (Additional file 1: Figure S3).

### Activated Akt signaling conveys resistance to cell death induced by chemotherapeutic drugs

**Figure 7:**
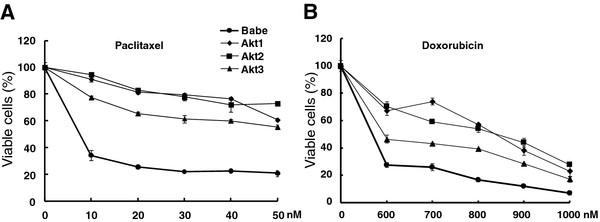
**Activated Akt signaling conveys resistance to chemotherapeutic drugs in an isoform redundant manner.** MCF10A cells were infected with retroviruses to respectively express empty vector (Babe), Akt1, Akt2 or Akt3 and then treated with various dosages of paclitaxel (**A**) and doxorubicin (**B**) for 2 and 5 days. % of viable cells was calculated by setting untreated groups as 100% to which the drug treated cells were compared. Akt-overexpression significantly sustained cell viability in response to treatments by cytotoxic agents (*p* < 0.05).

The findings presented above demonstrate that activated Akt renders either inhibitory or marginal, but never enhancing, effects on EMT transcripts, cell motility and in maintenance of stem/progenitor cell populations. These observations are paradoxical because they are opposite to the general oncogenic effects normally associated with Akt. To further delineate whether or not Akt activation can exert other tumor-promoting effects, we assessed if apoptotic death induced by two common chemotherapeutic agents, paclitaxel and doxorubicin, can be halted. As shown in Figure 7A and 7B, Akt activation dramatically augmented cell viability in response to drug treatment. This protective effect spans a broad range of doses (10-50 nM for paclitaxel and 600-900nM for doxorubicin), supporting a notion that Akt activation manifests its oncogenic effect minimally by preventing cells from apoptotic death induced by cytotoxic agents. Moreover, we showed that all 3 Akt isoforms exerted redundant, rather than distinct or opposing effects in maintaining cell viability.

## Discussion

A growing body of evidence has demonstrated that activation of components within the PI3K cascade are associated with human carcinomas including colon, endometrium, prostate, brain, ovarian, and breast cancers [15,48]. However, gain-of-function mutations leading to constitutive activation of Akt are relatively uncommon (E17K is the most frequent occurrence) [15,17], which contradicts a general belief that Akt activation plays critical roles in driving neoplastic phenotypes. In breast cancer, the oncogenic roles associated with Akt isoforms are still unclear and the discrepancies may be due to factors associated with mouse models vs. clinical studies, knockdown vs. overexpression, and *in vitro* vs. *in vivo*[27-30,32,49]. Akt1 has been shown to inhibit EMT as well as cell motility and these aberrations can be rescued by Akt2, so that the net balance and ratio between the two isoforms dictated the overall cell fate [27,29]. However, this observation raised two unresolved issues: (a) what upstream mediators, if any, would select which isoforms to be activated; and (b) what downstream targets can be uniquely transmitted in response to different isoforms and exert distinct and perhaps opposing effects. In the current report, we demonstrate that Akt isoforms appear to have redundant, rather than unique functions, when promoting neoplastic features.

We have identified Akt1 as being the major isoform in all breast epithelial cells examined in the current report (Additional file 1: Figure S4). In other independent studies, however, activation of Akt1 was demonstrated to suppress EMT [27], an event also important for stem cell self-renewal [23,29]. Taken together, these findings suggest that upregulated HER2 or knocked down PTEN would have not only activated Akt1 signaling but also repressed EMT and subsequently lowered stem/progenitor subfraction. However, this rationale is contradictory to data reported in previous publications [26,42]. Nevertheless, our present findings suggest an unreported paradigm that all Akt isoforms are likely to behave similarly for repressing cell migration, EMT, and stem/progenitor function, rather than exerting antagonistic effects by interacting among various isoforms. Two possibilities may explain the discrepancies between our data and others. Not only does the malignant state and cell context dictate Akt’s inhibitory effects (Figure 6), but the published findings are mainly generated from Akt knockdown studies that might induce unexpected feedback signaling. Notably, loss of Akt may not be physiologically relevant since most human carcinomas are associated with activation, rather than an inhibition, of Akt signaling. To date, Akt ablation has not been reported for any of the known human malignancies. Regarding isoform specificity, our current data cannot completely exclude the possibility that other undisclosed downstream targets or events might respond to Akt activation in an isoform-specific manner. However, this issue is beyond the scope of our current study.

The inhibition of cell migration and EMT by Myr-Akt in the current study appears to recapitulate some of the aberrations induced by *PIK3CA* mutations. This notion is supported by a clinical study from a large cohort of breast tumors (*N* = 590) in which the women who carried activation mutations of *PIK3C* also displayed improved prognosis, prolonged breast cancer-specific and overall survival as well as lymph node negativity [32]. However, our finding that Myr-Akt overexpression failed to expand stem/progenitor cell subpopulation is somewhat inconsistent with the data from exogenous expression of HER2 or from knockdown of *PTEN* by ShRNA [26,42]. We reason the discrepancy is likely due to the fact that dysregulated HER2 and PTEN can trigger far broader downstream targets beside Akt. For instance, other than acting as a phosphatase to attenuate activated Akt, PTEN can regulate cell cycle progression, stem cell self-renewal, chromosome stability, and senescence [50]. Likewise, additional signaling cascades downstream from PI3K include mitogen-activated protein kinase (MAPK), extracellular signal-related kinase (ERK), and Wnt/β-catenin, which may synergistically promote stem-progenitor self-renewal and override the inhibitory effect solely incurred from Akt. It is worthy to mention that PI3K signaling can lead to not only Akt-dependent but also Akt–independent activation [51] and the latter might be partly responsible for combating the inhibitory effect from the former.

The present report demonstrates that in spite of exerting tumor-suppressing effects, Akt can render an opposing oncogenic event by resisting cell death induced by the action of cytotoxic drugs. It thereby suggests that not only ectopically expressed Myr-Akt retained authentic functionality, but also that Akt signaling regulates pleotropic downstream substrates, each of which exerts distinct outcomes. For instance, Forkhead transcription factors (FOXO1), BAD, caspase 9, and NF-κB seem to be responsible for protecting cells from apoptosis, whereas mTOR and Wnt/β-catenin signaling might be involved in regulating stem/progenitor cells [23]. In support of this notion, we have observed that, besides repressed EMT and stem cell self-renewal, overexpresssion of Myr-Akt protected cells from apoptotic death induced by Paclitaxel as well as by Doxorubicin (Figure 7). Coincidently, our finding about resistance to apoptosis induced by Doxorubicin intervention can be supported by independent data generated from *in vitro* knockdown of *PTEN* in cell culture system [52].

## Conclusions

We present a novel paradigm that Akt activation can have dichotomous effects on neoplastic progression. Akt’s intrinsic property of tumor-suppressive effects is demonstrated by repression of EMT, cell motility, and stem/progenitor cell expansion, and that the effects are notably distinct from its tumor-promoting functions that enhance cell survival. While the former would confine the target cells (mainly at non- or low-malignant states) to the local sites, the latter partly contributes to its oncogenic effect. Restrained cells then await additional tumorigenic signals presumably provoked from tumor microenvironmental factors or from additional carcinogenic insults that would alleviate the tumor-suppressive effect of Akt prior to metastatic spread. Meanwhile, epithelial cells would be maintained in a viable state during the course of therapeutic drug treatments.

## Materials and methods

### Cell culture, retroviral infections and siRNA delivery

Normal human mammary epithelial cells (HMEC) derived from three different subjects were purchased from and authenticated by Lonza as well as ScienceCell Research Laboratories and cultured in mammary epithelial growth medium (MEGM) (Lonza). MCF10A, the spontaneously immortalized human normal epithelial cell line, was acquired from and authenticated by American Type Culture Collection (Manassas, VA). MCF10A1 (MI), MCF10AT1k.cl2 (MII) and MCF10CA1h (MIII) cells [47] were obtained from Barbara Ann Karmanos Cancer Institute (Detroit, MI) and grown in DMEM/F12 medium (Mediatech) supplemented with 5% horse serum, EGF (20 ηg/ml), insulin (10 μg/ml), hydrocortisone (500 ηg/ml), and cholera toxin (100 ηg/ml). The BrCa-MZ-01 and SUM159 breast cancer cells were generous gifts from Dr. Max S. Wicha (University of Michigan, Ann Arbor, Michigan) and are commercially available (Asterand, Detroit, MI). BrCa-MZ-01 cells were maintained in RPMI1640 supplemented with 10% Fetal Bovine Serum (FBS) whereas SUM159 was propagated in F12 medium (Mediatech) with 5% FBS, insulin (5 μg/ml), and hydrocortisone (1 μg/ml). Antibiotic-antimycotic (100 U/ml penicillin, 100 μg/ml streptomycin and 0.25 μg/ml amphoterincin) was routinely included in medium to prevent microbial contamination.

pBabe-Puro, pBabe-Puro-Myr-Flag-Akt1 [8], pBabe-Puro-Myr-HA-Akt2, pBabe-Puro-Myr-HA-Akt3, pBabe-Bleo, and pBabe-Bleo-IGF-1R, were purchased from Addgene Inc. To obtain infectious retrovirions prior to transducing Myr-Akt into target cells, retroviral vectors were first introduced into packaging cells known as Phoenix™ Ampho (Orbigen, Inc.) by a calcium phosphate transfection method. 24 hours later, the medium was replenished and the resultant supernatant was collected twice at 12-hour intervals and each harvest was immediately overlaid on the target cells. Afterwards, the infected cells were selected for with either 2.5 μg/ml puromycin (for pBabe-Puro vector) or with 500 μg/ml zeocin (for pBabe-Bleo backbone plasmids) for 7 days and the drug-resistant cells were then collected on the 14th day after infection.

FBS, horse serum, B27 serum-free supplement, basic fibroblast growth factor (bFGF) and zeocin were obtained from Invitrogen; EGF, cholera toxin, hydrocortisone, insulin, puromycin, paclitaxel and poly-HEMA were purchased from Sigma; and Doxorubicin was from Calbiochem.

For knocking down endogenous Akt expression using RNA interference (siRNA), IGF-1R-expressing MCF10A cells (transduced by pBabe-Bleo-IGF-1R retrovirus) were transfected in triplicate with Thermo Scientific Dharmacon ONTARGET*plus* siRNA SMARTpool reagents against individual or combinations of the Akt1 and Akt2 (Thermo Scientific Cat # L-003000-00 and L-003001-00, respectively) following the protocols recommended by the manufacturer (Thermo Scientific). To generate a negative control, cells were similarly transfected with the ON-TARGET*plus* Non-Targeting siRNA Pool (Thermo Scientific Cat # D-001810-10).

### Western blot analysis

Cells were lysed in NP-40 lysis reagent (1% NP-40, 50 mM Tris, pH 7.6, and 150 mM NaCl) or in RIPA lysis buffer (Cell signaling) supplemented with protease inhibitor cocktail tablets (Roche). 30-50 μg of proteins were resolved by 8-10% SDS-PAGE and immunoblotted using standard techniques. Antibodies recognizing phosphorylated Akt (Ser 473), N-cadherin, Akt (pan), Akt1, Akt2, Akt3, GAPDH, phosphorylated and pan IGF-1R were obtained from Cell Signaling Technology whereas the antibodies respectively recognizing E-cadherin, fibronectin, and vimentin were purchased from BD Biosciences.

### Reverse transcription followed by quantitative real-time PCR analysis (RT-qPCR)

Total RNA was extracted with Trizol (Invitrogen) and 1.0 μg of which served as the templates for synthesizing complementary DNA (cDNA) by using SuperScript III reverse transcriptase (Invitrogen). The resultant cDNA together with the RT^2^ SYBR Green qPCR Master Mixes (SABiosciences) were used for real-time PCR analysis on a 7500 fast real-time PCR machine (Applied Biosystems). Quantification of mRNA expression was normalized to the internal transcript of GAPDH. The primers used for GAPDH were 5’-CCCCTTCATTGACCTCAACTACAT-3’ (forward) and 5’-CACTCCTGGAAGA TGGTGA-3’ (reverse). Other primers utilized for amplification of EMT-associated transcripts were described previously [23].

### Transwell migration assay

Cells obtained from sub-confluent culture were dissociated by trypsinization and resuspended in limiting culture medium containing a reduced concentration of serum or devoid of bovine pituitary extract (BPE). 2-5 × 10^4^ of the resultant cells were loaded into the top chambers of 24-well transwell plates (8 μm pore size; BD Biosciences) whereas the bottom chambers were filled with only regular culture medium without any cells. About 12-24 hours later, the non-motile cells at the top of the filter were swapped off with cotton swabs while the motile cells at the bottom of the filter were fixed with 70% ethanol and stained with 0.1% crystal violet. The number of migrated cells was quantified by the counting of 10 fields under 20X magnification of a microscope to generate an average value.

### Wound healing assay

Approximately 5 × 10^4^ cells were plated into each well of 6-well plates and treated with either vehicle control or with 2 ηg/ml TGFβ (R & D Systems) for 3 days. On the day of the experiment, the monolayer of confluent cells was lightly scratched with a pipette tip and photographed immediately and 20 hours later. The ability of healing the scratched wound was accessed using ImageJ software by calculating % of decreased scratch area at 20 hours in relation to the one at 0 hour. At least 10 scratch areas were scored to generate an average value.

### Flow cytometry

Cells were trypsinized, washed once with ice-cold stain/wash buffer (HBSS + 2% FBS), and then resuspended at a concentration of 1 × 10^7^/ml in which specific antibody was added. Cells were incubated for an additional 20-30 minutes on ice, washed twice with the same buffer and then subject to flow cytometry analysis using BD FACSAria Flow Cytometer (BD Biosciences). The antibodies recognizing PE-CD24 (clone ML5), FITC-CD44 (clone G44-26), and their isotype controls were obtained from BD Biosciences. During the flow cytometric analysis, the vast majority of intact cells were gated based on forward and side scattering plots. Cells stained with PE- normal IgG and FITC- normal IgG were used to set up respective gates for PE-CD24 and FITC-CD44 single staining. The resultant baselines subsequently generated the combinatorial gate for quantifying CD24 plus CD44 doubly stained cells (Additional file 1: Figure S5). The cells displaying CD44^+^/CD24^-/low^ were regarded as the subpopulation enriched with stem/progenitor cells (Additional file 1: Figure S5F).

### ALDEFLUOR assay

The ALDEFLUOR kit (StemCell Technologies) was employed to quantify the subpopulation of cells with a high ALDH enzymatic activity. Briefly, cells were trypsinized, resuspended at a concentration of 1 × 10^6^/ml in ALDEFLUOR assay buffer containing ALDH substrate (BAAA), and incubated for an additional 30-60 minutes at 37°C. To generate a cell faction representing a base-line negative control, a small aliquot of the respective sample was incubated with DEAB (a specific ALDH inhibitor) immediately after the addition of BAAA. After incubation, stained cells were washed with ice-cold wash buffer (1 × HBSS + 2% FBS) twice and then subject to flow cytometry analysis as previously described [53]. Cells harboring ALDH activity higher than the baseline level (upper panels of Additional file 1: Figures S3A and S3B) were scored as an ALDH^+^ subfraction that is enriched for stem/progenitor cells.

### Mammosphere assay

Growth of mammospheres was carried out as previously described [54]. Briefly, single cell suspensions at a density of 1-5 × 10^3^/ml were plated in serum-free mammary epithelial basal medium (MEBM) (Lonza) supplemented with B27, EGF (30 ηg/ml), bFGF (20 ηg/ml), insulin (5 μg/ml), hydrocortisone (0.5 μg/ml), heparin (4 μg/ml), gentamycin (4 μg/ml) and antibiotic-antimycotic in poly-HEMA-coated culture dishes. 10-14 days later, the number of mammospheres with sizes of ≥70 μm were either counted or further dissociated into another single-cell suspensions and grown for a subsequent passage assay.

### Survival assay

Approximately 8,000 cells at their exponential growth stage were seeded in each well of 96-well plates in triplicates. On the next day, cells were treated with various concentrations of doxorubicin or paclitaxel for 2 or 5 days, and then viable cells were quantified using MTT.

### Statistical analysis

The Student’s *t* test was carried out to assess if the differences between experimental samples to the control were significant (indicated by *p* < 0.05).

## Abbreviations

E-cad: E-cadherin; EMT: Epithelial-mesenchymal transition; FBS: Fetal bovine serum; FN1: Fibronectin; HMEC: Human mammary epithelial cells; N-cad: N-cadherin; PTEN: Phosphatase and tensin homolog; VIM: Vimentin.

## Competing interests

The authors declare that they have no competing interests.

## Authors’ contributions

HL initiated, designed the studies, and oversaw the completion of the project. ZP conducted the vast majority of experiments. Briefly, ZP, JCW, JRS, ZH and WZ performed viral transduction studies, EMT analysis, Western blots, transwell migration, cell viability, flow cytometry and other related experiments. RS, and MC provided clinical, pathological, or scientific insights of breast cancer. All authors helped in discussing, reading and proofreading the final manuscript. All authors read and approved the final manuscript.

## Supplementary Material

Additional file 1 Dichotomy Effects of Akt Signal on Breast Neoplasm. Click here for file
