# Supplementary material for: Dichotomy effects of Akt signaling in breast cancer
Source: Mol Cancer. 2012 Aug 24;11:61. doi: 10.1186/1476-4598-11-61 (PMC3494580; doi:10.1186/1476-4598-11-61)
Supplement: Additional file 1 — Dichotomy Effects of Akt Signal on Breast Neoplasm. [file 1476-4598-11-61-S1.doc]

**SUPPLEMENTAL DATA**

**Dichotomy Effects of Akt Signal on Breast Neoplasm**

Zhengang Peng, Jennifer Chao Weber, Zhaosheng Han, Rulong Shen, Wenchao Zhou, James R. Scott, Michael W.Y. Chan, and Huey-Jen L. Lin


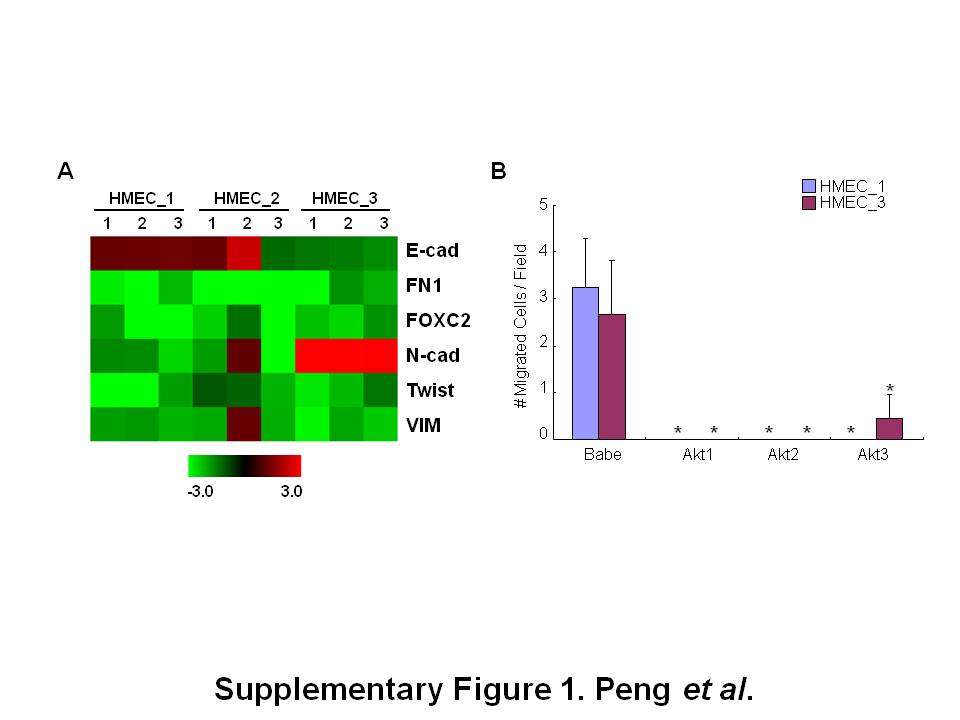


**Supplementary Figure 1.** Exogenous expression of Akt, regardless of isoform types, inhibits migration of HMEC cells**.** HMEC#1 and HMEC#3 cells were infected with retroviruses respectively express control (Babe), Myr-Akt1 (1), Akt2(2) or Akt3 (3) at two weeks prior to experiments started. Resultant cells were wither assessed for the relative expression of EMT-associated transcripts (A) or seeded on inside chambers of transwell plate and then assessed by migration assay after 24 hours (B). * represents a significant reduction of migrated cells in Akt-expressing cells, as compare to Babe group (if p<0.05).


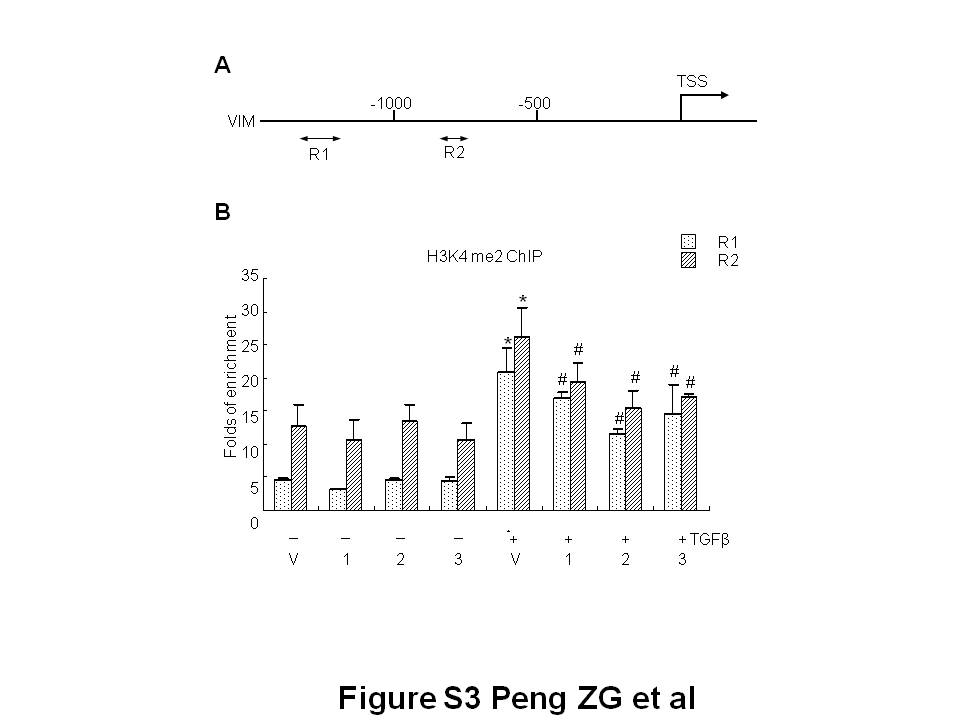


**Supplementary Figure 2.** Activated Akt signaling attenuates H3K4me2 occupancy on the *VIM* promoter after TGFβ treatment, a phenomenon might convey a possible epigenetic mechanism denoting how activated Akt silences VIM as well as impedes EMT induced by TGFβ. MCF10A cells were first infected with retroviruses to express BABE vector control (V) or Myr-Akt1(1), Akt2 (2) and Akt3 (3). Cells were then either left untreated or treated with TGFβ. Histone occupancy at the promoter region was carried out by chromatin immunoprecipitation (ChIP) as described previously (Molecular Cell 2006;21:393–404). Briefly, cells were fixed with 1% formaldehyde that cross-links proteins (histones) to DNA. The resultant DNA-protein complexes were sonicated followed by immunoprecipitation using Dynabeads Protein G (Invitrogen) coated with H3K4 me2 antibody (Upstate). The DNA fragments were later dissociated from the aforementioned immunocomplexes and the concentration was accurately determined by PicoGreen dsDNA Assay kit (Invitrogen). The resultant pulled-down DNA, together with the input DNA without ChIP enrichment, was subject to qPCR analysis by using specific primers interrogating the *VIM* promoter region. While TSS denotes transcriptional-start site, the schematic diagram of *VIM* promoter illustrates the locations of R1 and R2 in relation to TSS. The primers utilized for ChIP-qPCR are R1: 5’-ccgccaaagattctgtcatt-3’ (forward) and 5’-gtggtttttaccctggtgga-3’ (reverse); R2: 5’-ggcccagctgtaagttggta-3’ (forward) and 5’-cctagcggtttaggggaaac-3’ (reverse) (A). Folds of enrichment were calculated by comparing to input DNA that serves as a control. A representative data was generated from three independent experiments (B). ^*^ denotes significant changes (if p<0.05) by comparing data generate from the vector control cells (V) treated with TGFβ to the one derived from untreated cells. Likewise, # represents significant changes derived from a comparison of the Akt-expressing cells treated with TGFβ to the one from counterpart vector control cells under the same condition (if p<0.05).


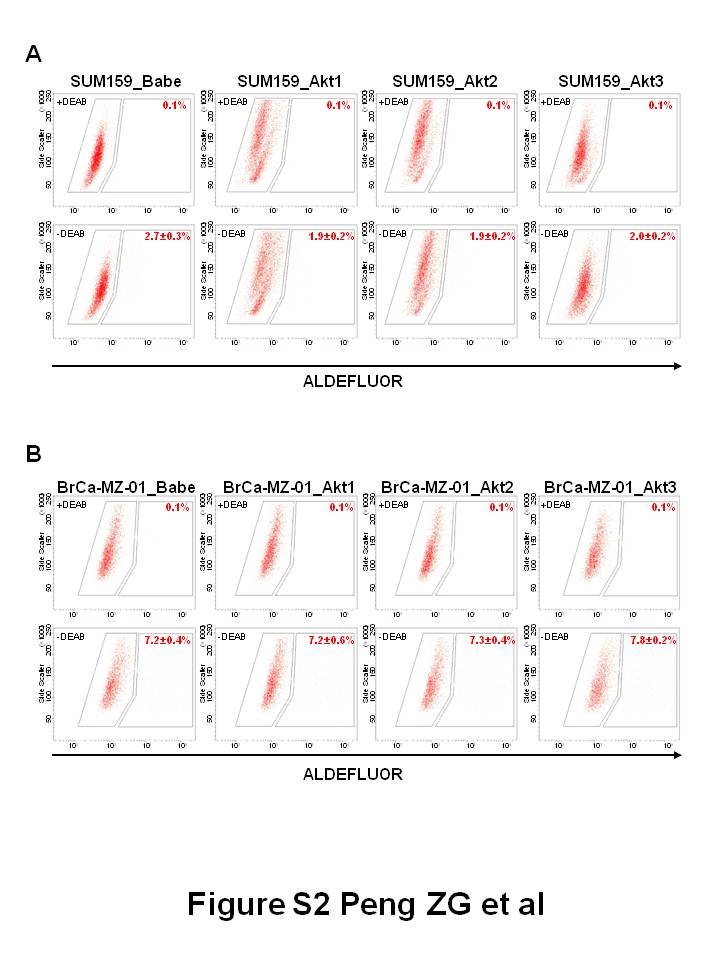


**Supplementary Figure 3.** Activated Akt signaling renders negligible influence on stem cell subpopulation in breast cancer cell lines. Two representative lines SUM159 **(A)** and BrCa-MZ-01 **(B)** were respectively infected with either control (Babe), or with Myr-Akt-expressing retrovirus namely Akt1, Akt2 or Akt3. The stem/progenitor cells within the resultant cells were then quantified by ALDEFLUOR assay. Upper panel denoted cells were pre-treated with inhibitor DEAB so that they serve as negative controls for quantifying ALDEFLUOR-positive fractions (cells designated as –DEAB and gathered in the lower panel) that reflect stem cell-enriched subfractions. Data is a representative from three independent experiments.


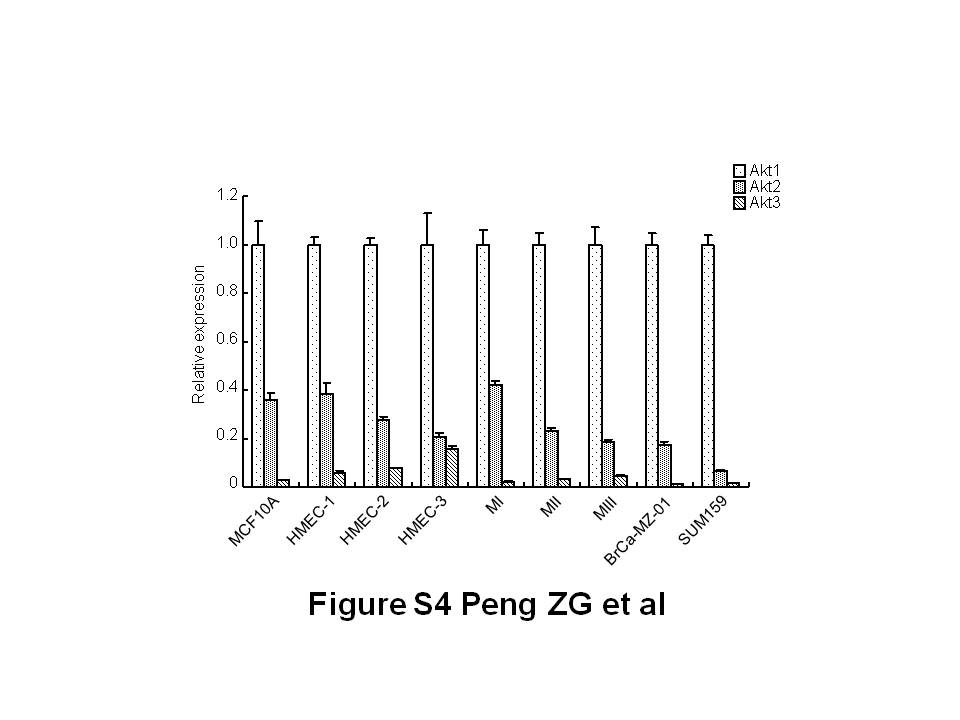


**Supplementary Figure 4.** Akt1 is the predominant isoform expressed in the breast epithelial cells studied in this report. The total RNA was extracted from indicated cells, followed by RT-qPCR analysis to quantify the levels of endogenous Akt isoforms. In each cell line, the expression of Akt1 was compared to the one from GAPDH and the ratio was set as being 1, to which the relative expression of Akt2 and Akt3 were compared.

**
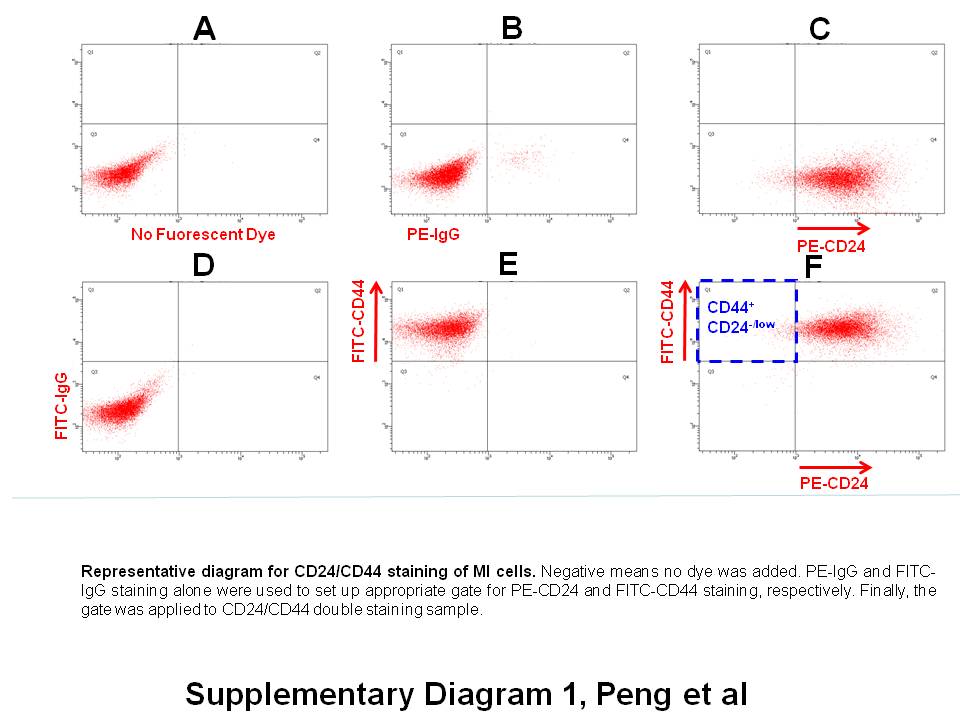
**

**Supplementary Figure 5.** Representative dot plots derived from flowcytometric analysis of MI cells. To set baseline controls, cells were either untreated to show the auto-fluorescent cells (A), or stained with normal IgG isotype control antibodies conjugated with fluorescent dye PE (B) or FITC (D). Next, another aliquot of cells were stained with specific antibodies PE-CD24 (C) or with FITC-CD44 (E) or in combination. Cells harboring fluorescent signal greater than the base-line controls are scored as positive sub-fraction and the ones displaying CD44^+^plus CD24^-/low^ properties are regarded as the subpopulation enriched with stem/progenitor cells (outlined by blue dashed lines in panel F).
